# Long‐term stability and reproducibility of frozen plasma control samples for hepatitis B virus, hepatitis C virus, and human immunodeficiency virus by quantitative PCR: Implications for retrospective testing in hemovigilance

**DOI:** 10.1111/trf.70398

**Published:** 2026-09-19

**Authors:** Benjamin Marx, Annemarie Berger, Niko Kohmer, Marhild Kortenbusch, Sebastian Hoehl, Sandra Ciesek, Holger F. Rabenau

**Affiliations:** ^1^ Institute for Medical Virology University Hospital Frankfurt, Goethe University Frankfurt Frankfurt am Main Germany; ^2^ Fraunhofer Institute for Translational Medicine and Pharmacology (ITMP) Frankfurt am Main Germany

**Keywords:** frozen plasma, hemovigilance, hepatitis B virus, hepatitis C virus, human immunodeficiency virus, nucleic acid stability, quantitative PCR

## Abstract

**Objective:**

Data on the long‐term stability and reproducibility of nucleic acid amplification testing (NAT) results in frozen stored positive samples (≤−20°C) remain limited. This study evaluated the long‐term performance of frozen virus‐positive samples in quantitative PCR assays and aimed to provide evidence for their analytical reliability in retrospective NAT and quality assurance. Because blood transfusions, despite their high safety, still carry a residual risk of transmission of blood‐borne viruses, these data may also be relevant for look‐back investigations in hemovigilance.

**Materials and Methods:**

Diluted and aliquoted internal run controls from positive patient samples were retrospectively analyzed using routine quantitative PCR assays. Long‐term stability was assessed over 37 months for HIV, 25 and 29 months for HBV‐A and HBV‐B, and 49 and 38 months for HCV‐A and HCV‐B. Descriptive statistics and linear regression were used to assess variability and temporal trends. Compliance with the German Medical Association's guidelines for quality assurance in medical laboratory testing (Rili‐BAEK) was evaluated using an acceptance range of ±0.5 log10.

**Results:**

All internal run controls demonstrated stable long‐term performance during frozen storage and routine use. Across the datasets, 99.2%–100% of measurements remained within the acceptance range. Results outside mean ± 3 SD were rare, and no statistically significant temporal trend was observed for any internal run control.

**Conclusion:**

Frozen HBV‐, HCV‐, and HIV‐positive samples showed robust long‐term stability and reproducibility in routine quantitative PCR assays for up to 4 years. These findings support the suitability of long‐term stored specimens for retrospective NAT.

**Abbreviations**
HBV: hepatitis B virus
HCV: hepatitis C virus
HIV: human immunodeficiency virus
IRC: internal run control
NAT: nucleic acid amplification testing

## 1 INTRODUCTION

Blood transfusion represents an essential component of modern healthcare and is highly safe in high‐income countries. Nevertheless, transfusion‐transmitted infections caused by hepatitis B virus (HBV), hepatitis C virus (HCV), or human immunodeficiency virus (HIV) remain a relevant safety concern, as these infections may lead to chronic disease and, in the case of HIV, lifelong treatment. Nucleic acid amplification testing (NAT) has been widely implemented in blood screening in most high‐income countries and is increasingly being adopted in middle‐income settings.^1^ By enabling direct detection of viral genomes during the serological window period, before antibodies become detectable, NAT has substantially reduced the residual risk of transfusion‐transmitted infection. Several studies have shown that NAT substantially reduces the residual risk compared with serology alone, with an estimated reduction of approximately 49% for HBV and more than 94% for HCV. For HIV, the additional reduction in residual risk is smaller but still clinically relevant and largely reflects the shortening of the diagnostic window period, depending on the screening setting and assay strategy.^2^, ^3^, ^4^, ^5^, ^6^ Consequently, confirmed transfusion‐transmitted infections have become rare. In Germany, suspected cases of transfusion‐transmitted HBV and HCV infection continue to be reported within the hemovigilance system, whereas no transfusion‐associated HIV, HCV, or HBV transmission was confirmed in the 2022 reporting period.^7^

In Germany, traceability requirements mandate the retention of blood donation samples for future investigations, including epidemiological analyses, quality assurance, and retrospective investigations (look‐back investigations) following suspected transfusion‐related adverse events. These activities are supported by national provisions for sample retention. According to the recommendations of the German Advisory Committee Blood, follow‐up samples must be retained for at least 3 years or for at least 1 year beyond the shelf life of the plasma prepared from the respective donation.^8^ Such retained samples are a key component of hemovigilance systems, which aim to detect, investigate, and prevent transfusion‐related complications. Although suspected cases continue to be reported, only a small proportion are ultimately confirmed in countries with established donor screening and surveillance systems.

Despite these advances, published data on the long‐term quantitative stability of frozen virus‐positive plasma material in routine NAT settings remain limited and are largely restricted to selected targets or pre‐analytical conditions.^9^, ^10^, ^11^ In particular, there is little information on how repeatedly tested frozen positive control material performs over extended storage periods under real‐world laboratory conditions. Degradation of RNA or DNA during prolonged storage could potentially impair detection or bias quantitative measurements, thereby limiting the validity of retrospective investigations and long‐term quality assurance. In addition to storage duration, factors such as storage temperature, freezing conditions, and the type of storage may also influence nucleic acid stability. The present study therefore evaluated the long‐term stability and reproducibility of frozen HBV‐, HCV‐, and HIV‐positive internal run controls (IRCs) in routine quantitative PCR assays. By assessing longitudinal quantitative results over extended storage periods, this study aimed to provide supportive evidence for the analytical reliability of long‐term frozen virus‐positive plasma material in retrospective NAT and quality assurance, including applications in hemovigilance.

## 2 MATERIALS AND METHODS

### 2.1 Study design and selection of observation periods

To assess the long‐term stability of the IRCs used in routine quantitative PCR testing for HIV, HBV, and HCV, we performed a retrospective analysis of quantitative PCR results generated during routine diagnostic testing. All measurements were generated using the COBAS AmpliPrep/COBAS TaqMan platform, as described in detail below. In addition to the manufacturer‐provided positive and negative controls routinely included in each assay run, we implemented a manufacturer‐independent IRC, which was likewise analyzed in each run. Only observation periods during which the same IRC preparation was used continuously for internal quality control were included. For HIV, one IRC preparation was evaluated over 968 measurements and a storage period of 37 months. For HBV, two consecutive IRC preparations were analyzed: HBV‐A over 308 measurements and 25 months, and HBV‐B over 340 measurements and 29 months. For HCV, two IRC preparations were evaluated: HCV‐A over 774 measurements and 49 months, and HCV‐B over 183 measurements and 38 months.

### 2.2 Preparation of internal run controls

Each IRC was prepared from a highly positive individual patient sample diluted in plasma that had tested negative for HIV, HBV, and HCV by NAT. The initial concentration of the source material was at least 4.2 × 10^6^ copies/mL for HIV, 2.5 × 10^6^ IU/mL for HBV‐A and HBV‐B, and 3 × 10^7^ IU/mL for HCV‐A and HCV‐B. Separate IRC preparations were produced for each virus.

After dilution to the intended target concentration of 10^2^–10^3^ copies/mL or IU/mL, the respective suspensions were prepared in 2‐L glass bottles and homogenized using a magnetic stirrer for approximately 10 min. One aliquot from each preparation was then pretested using the same quantitative PCR platform and assay principle described below to confirm that the measured concentration corresponded to the expected target value. Following preparation, 1 mL aliquots of each IRC were dispensed into cobas input tubes (Roche Diagnostics, Mannheim, Germany; P/N 03137040001), individually sealed, and stored frozen at ≤−18°C. Storage temperature was monitored daily using a minimum–maximum thermometer.

### 2.3 Initial characterization of the IRC preparations

To determine the initial target value and evaluate assay reproducibility, 20 quantitative PCR measurements were performed during initial characterization for each IRC preparation representing HIV, HBV, or HCV. The mean of these first 20 characterization measurements was defined as mean1 and served as the reference value for subsequent assessment according to the German guideline for quality assurance in laboratory medicine (Rili‐BAEK).^12^ These measurements were performed under the standard assay setup used at the time, including triplicate measurements and/or single measurements across different runs, in line with general principles of assay verification and validation in clinical virology and diagnostic laboratory practice.^13^, ^14^ According to the available dataset, these initial measurements were included in the subsequent longitudinal analysis.

### 2.4 Virological testing

Quantitative real‐time PCR testing for HIV, HBV, and HCV was performed using the COBAS AmpliPrep/COBAS TaqMan (CAP/CTM; Roche Diagnostics, Mannheim, Germany) platform according to the manufacturer's instructions. HIV results are reported in copies/mL, whereas HBV and HCV results are reported in IU/mL. The assay versions and manufacturer‐provided analytical characteristics are summarized in Table 1. The IRCs were prepared to achieve target concentrations in the lower quantitative range of the respective assays (approximately 10^2^–10^3^ copies/mL or IU/mL), that is, clearly above the manufacturer‐reported LOD/LLOQ but within a range in which proportionally greater analytical variation can be expected.

**TABLE 1:** Manufacturer‐provided characteristics of the quantitative CAP/CTM assays used for HCV, HBV, and HIV testing.

Parameter	HCV CAP/CTM v2.0	HBV CAP/CTM v2.0	HIV‐1 CAP/CTM v2.0
Input volume (mL)	0.8	0.5	0.8
Target region	5′ UTR	Precore/Core region	gag/LTR
LLOQ/LOD	15 IU/mL	20 IU/mL	20 copies/mL

*Note*: HIV results are expressed in copies/mL, and HBV and HCV results in IU/mL.

Abbreviations: CAP/CTM, COBAS AmpliPrep/COBAS TaqMan; LLOQ, lower limit of quantification; LOD, limit of detection; IU, international units.

### 2.5 Statistical analysis

IRC measurement results were analyzed separately for each virus and IRC preparation. Descriptive statistics included the mean, standard deviation (SD), minimum, maximum, and coefficient of variation.

Temporal trends during storage were assessed by simple linear regression of raw quantitative measurement results over time. This approach was chosen as a pragmatic assessment of potential absolute drift during routine use, whereas predefined acceptance limits were evaluated separately on a log10 scale according to Rili‐BAEK criteria. The slope of the regression line was determined and evaluated to identify any significant deviation from zero.

In addition, the proportions of results outside mean ± 2 SD and mean ± 3 SD were recorded as descriptive indicators of analytical variability based on all measurements within the respective dataset. These parameters were used for descriptive assessment only and not as formal acceptance criteria.

For internal quality assurance, results were also evaluated according to Rili‐BAEK.^12^ Compliance was assessed using the acceptance range mean1 ± 0.5 log10. This criterion served as the primary benchmark for long‐term performance.

All statistical analyses and graphical evaluations were performed using GraphPad Prism version 10.2.0 (GraphPad Software, San Diego, CA, USA).

## 3 RESULTS

Quantitative HIV results are expressed in copies/mL, whereas HBV and HCV results are expressed in IU/mL.

### 3.1 HIV

A total of 968 quantitative PCR measurements of the HIV IRC obtained during routine use over 37 months were included in the analysis. All results remained within the predefined acceptance range of mean1 ± 0.5 log10. In addition, 5.5% of measurements were outside mean ± 2 SD, whereas only 0.7% were outside mean ± 3 SD.

Across the observation period, the distribution of HIV measurements remained overall consistent, with no evidence of a relevant loss of signal during storage. Linear regression analysis showed no statistically significant temporal trend in measured HIV viral load. The coefficient of variation was 26.2%.

In summary, the HIV IRC showed stable long‐term performance over 37 months of storage and routine use (Table 2, Figure 1).

**TABLE 2:** Descriptive statistics of the IRC datasets.

	HIV	HBV (A)	HBV (B)	HCV (A)	HCV (B)
Months	37	25	29	49	38
Number of values	968	308	340	774	183
Lowest value	314	174	518	95	42
Highest value	1780	2060	1770	1170	275
Range	1466	1886	1252	1075	233
Geometric mean	777	471	1028	344	134
Mean (all measurements)	807	502	1048	383	142
Mean1 (of first 20 measurements)	787	454	1162	452	138
Standard deviation	211	210	202	182	48
Coefficient of variation	26.2%	41.8%	19.3%	47.5%	33.4%
Number of values >mean + 2 SD	37 (3.8%)	7 (2.3%)	10 (2.9%)	44 (5.7%)	6 (3.3%)
Number of values <mean − 2 SD	16 (1.7%)	0 (0.0%)	8 (2.4%)	0 (0.0%)	1 (0.6%)
Number of values >mean + 3 SD	6 (0.6%)	5 (1.6%)	3 (0.9%)	9 (1.2%)	0 (0.0%)
Number of values <mean − 3 SD	1 (0.1%)	N/A	0 (0.0%)	N/A	N/A
Number of values >mean1 + 0.5 log10	0 (0.0%)	2 (0.7%)	0 (0.0%)	0 (0.0%)	0 (0.0%)
Number of values <mean1 − 0.5 log10	0 (0.0%)	0 (0.0%)	0 (0.0%)	6 (0.8%)	1 (0.6%)

*Note*: Values refer to copies/mL for HIV and IU/mL for HBV and HCV. Percentages outside mean ± 2 SD and mean ± 3 SD are reported as descriptive measures of variability based on all measurements of the respective dataset. Compliance with Rili‐BAEK requirements was assessed separately using mean1 ± 0.5 log10. N/A, not applicable. Mean1: mean of the first 20 characterization measurements of the respective IRC dataset.

**FIGURE 1:**
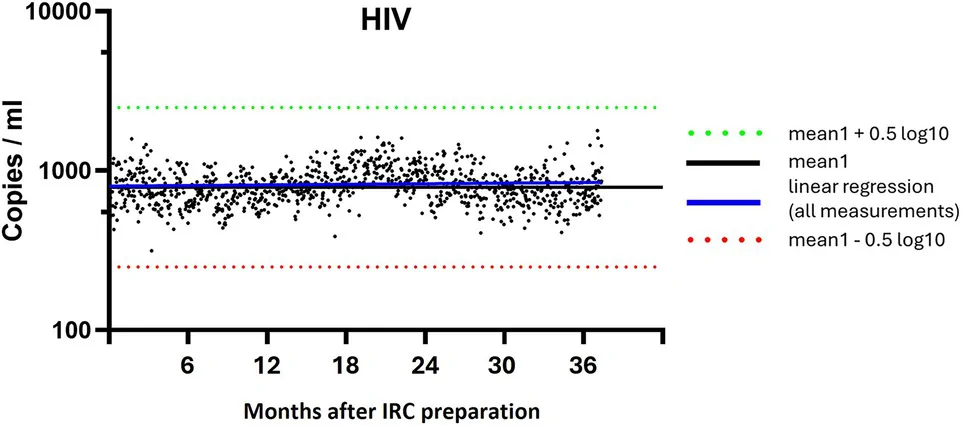
Long‐term performance of the HIV IRC. Longitudinal quantitative PCR results of the HIV IRC obtained during routine use over 37 months (968 measurements). Each dot represents one PCR measurement. Viral load is shown in copies/mL on a logarithmic scale. The black horizontal line indicates mean1, defined as the mean of the first 20 characterization measurements; the dotted lines indicate mean1 ± 0.5 log10, and the blue line represents the linear regression based on all measurements.

### 3.2 HBV

Two HBV IRCs, designated HBV‐A and HBV‐B, were evaluated over 25 months and 29 months, comprising 308 and 340 quantitative PCR measurements, respectively. For HBV‐A, 2 of 308 results (0.7%) were outside the acceptance range defined by mean1 ± 0.5 log10, whereas all HBV‐B results remained within this range. The proportion of results outside mean ± 2 SD was 2.3% for HBV‐A and 5.3% for HBV‐B. Five HBV‐A results (1.6%) and three HBV‐B results (0.9%) were outside mean ± 3 SD.

Over the observation period, both HBV IRCs showed stable longitudinal performance during storage and routine use. HBV‐A displayed broader scatter than HBV‐B, as reflected by coefficients of variation of 41.8% and 19.3%, respectively. Although a slight upward tendency was visually apparent for HBV‐A, linear regression analysis showed no statistically significant temporal trend for either HBV‐A or HBV‐B.

Taken together, both HBV IRCs demonstrated robust long‐term suitability for internal quality control over 25 and 29 months, respectively (Table 2, Figure 2).

**FIGURE 2:**
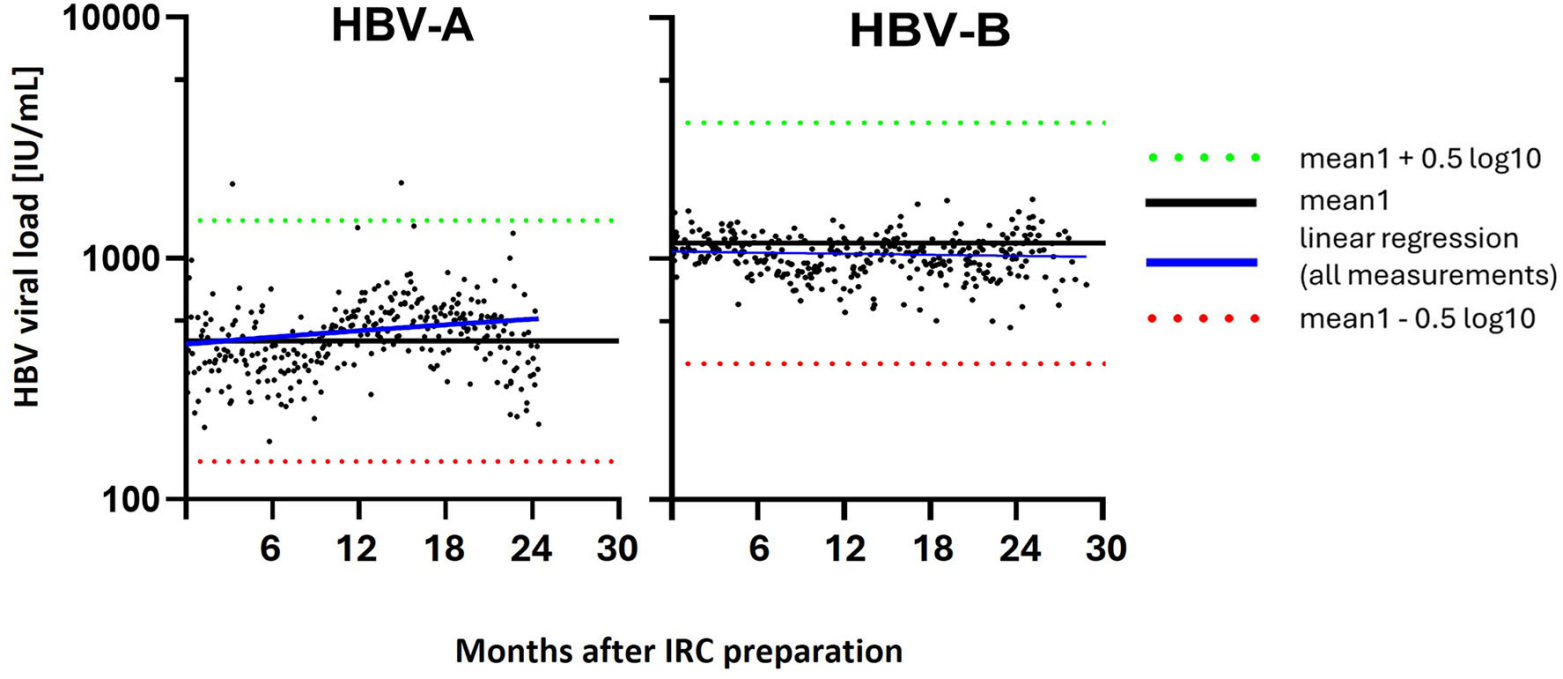
Long‐term performance of the HBV IRCs. Longitudinal quantitative PCR results of the two HBV IRCs, HBV‐A and HBV‐B, obtained during routine use over 25 months (308 measurements) and 29 months (340 measurements), respectively. Each dot represents one PCR measurement. Viral load is shown in IU/mL on a logarithmic scale. The black horizontal line indicates mean1, defined as the mean of the first 20 characterization measurements; the dotted lines indicate mean1 ± 0.5 log10, and the blue line represents the linear regression based on all measurements.

### 3.3 HCV

Two HCV IRCs, designated HCV‐A and HCV‐B, were evaluated over 49 months and 38 months, comprising 774 and 183 quantitative PCR measurements, respectively. For HCV‐A, 6 results (0.8%) were outside the acceptance range defined by mean1 ± 0.5 log10, and for HCV‐B, 1 of 183 results (0.6%) was outside this range. The proportion of results outside mean ± 2 SD was 5.7% for HCV‐A and 3.8% for HCV‐B. A total of 1.2% of HCV‐A results were outside mean ± 3 SD, whereas none of the HCV‐B results fell outside this range. Over the observation period, both HCV IRCs showed overall stable longitudinal performance during storage and routine use. HCV‐A displayed broader scatter and a slight visual downward tendency over time, whereas HCV‐B showed a narrower distribution with no relevant temporal change. This was reflected in coefficients of variation of 47.5% for HCV‐A and 33.4% for HCV‐B. Although HCV‐A showed a slight visual downward trend, this finding was neither statistically significant nor associated with reduced compliance with the predefined acceptance criteria. HCV‐B showed a slight visual upward tendency, but linear regression analysis likewise showed no statistically significant temporal trend.

Taken together, both HCV IRCs demonstrated robust long‐term suitability for internal quality control over 49 and 38 months, respectively (Table 2, Figure 3).

**FIGURE 3:**
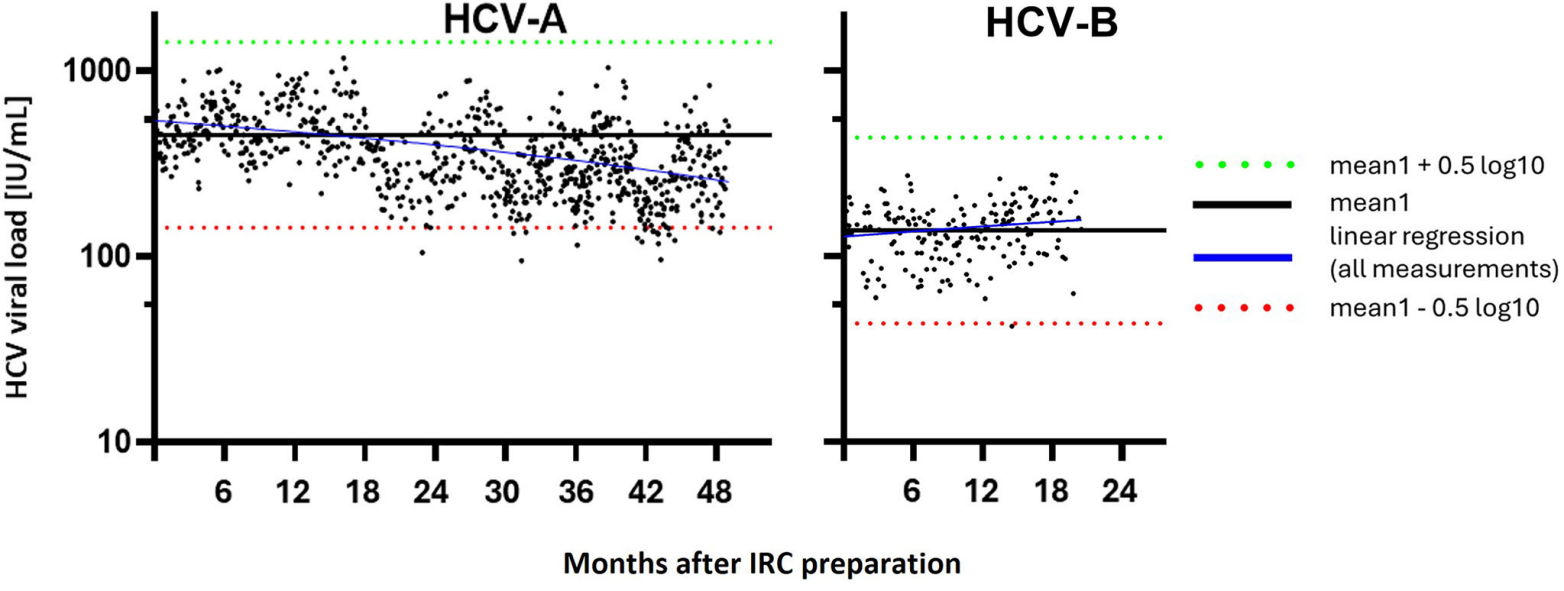
Long‐term performance of the HCV IRCs. Longitudinal quantitative PCR results of the two HCV IRCs, HCV‐A and HCV‐B, obtained during routine use over 49 months (774 measurements) and 38 months (183 measurements), respectively. Each dot represents one PCR measurement. Viral load is shown in IU/mL on a logarithmic scale. The black horizontal line indicates mean1, defined as the mean of the first 20 characterization measurements; the dotted lines indicate mean1 ± 0.5 log10, and the blue line represents the linear regression based on all measurements.

Overall, the IRCs demonstrated robust long‐term suitability for internal quality control during routine use (Figures 1, 2, 3, Table 2). The primary performance criterion was compliance with the Rili‐BAEK^12^ acceptance range defined by mean1 ± 0.5 log10, which was fulfilled by 99.2%–100% of all measurements across the different datasets. In addition, the low proportions of results outside mean ± 2 SD and mean ± 3 SD indicate limited analytical variability over time.

## 4 DISCUSSION

This study assessed the long‐term stability and reproducibility of frozen HBV‐, HCV‐, and HIV‐positive internal run controls used in routine quantitative PCR assays over periods of up to 4 years (49 months). Overall, the results indicate a high degree of longitudinal stability across all analytes. Nearly all measurements remained within the Rili‐BAEK acceptance range of mean1 ± 0.5 log10, and linear regression analysis showed no statistically significant temporal trend for any IRC. These findings support the assumption that frozen virus‐positive plasma samples can remain suitable for retrospective NAT, including look‐back investigations, over several years when stored and handled under controlled routine laboratory conditions.

The principal finding of this study is that prolonged frozen storage was not associated with a relevant loss of signal or with a systematic drift in quantitative PCR results. This is consistent with previous reports showing that viral RNA can remain detectable during frozen storage when pre‐analytical conditions are adequately controlled.^9^, ^10^ Although storage conditions differed between studies, these reports are broadly consistent with the present findings in supporting preservation of viral nucleic acid detectability under frozen storage. In molecular diagnostics, storage‐related effects are known to depend not only on storage duration but also on temperature stability, sample matrix, target concentration, and handling before analysis.^11^ Against this background, the present data provide supportive longitudinal evidence from routine laboratory practice rather than from a short‐term experimental setting.

The slight visual downward tendency observed for HCV‐A deserves consideration, although it was neither statistically significant nor associated with a relevant loss of compliance with the predefined acceptance criteria. One possible explanation is limited degradation of viral RNA over time, as RNA is generally considered more susceptible to pre‐analytical influences than DNA.^10^, ^11^ However, this interpretation remains tentative because no similar decline was observed for HCV‐B or the HIV IRC, both of which also contain RNA targets, although the shorter observation period and smaller dataset for HCV‐B should be taken into account. In addition, assay‐related influences such as reagent lot‐to‐lot variability cannot be excluded, because such factors were not systematically documented in this retrospective dataset. It is therefore possible that the pattern observed for HCV‐A reflects a combination of routine analytical variation and preparation‐related effects rather than a true storage‐related loss of viral RNA. Because the IRCs were generated by dilution of highly positive source material in negative plasma, subtle matrix‐ or dilution‐related effects may also have contributed. Taken together, the data do not indicate clinically or technically relevant progressive deterioration of HCV RNA during the observation period. The comparison between RNA viruses (HCV and HIV) and the DNA virus HBV is also informative. Although HBV DNA might theoretically be expected to be less susceptible to degradation than RNA genomes, the present study did not show clearly inferior long‐term performance of the RNA‐containing IRCs. HIV remained stable over 37 months, and both HCV IRCs also showed acceptable long‐term performance. This observation supports the view that, when plasma samples are stored frozen and unnecessary pre‐analytical stress is avoided, RNA stability may be sufficient for reliable retrospective NAT over extended periods.

At the same time, the higher coefficients of variation observed for some HCV and HBV preparations underline that assay‐ and batch‐specific variability must be considered when interpreting longitudinal control data and should not automatically be attributed to storage‐related degradation. The comparatively high coefficients of variation likely reflect measurements performed close to the lower quantitative range, where proportional analytical variation is generally expected to be greater. Importantly, despite this expected variability, nearly all measurements remained within the predefined Rili‐BAEK acceptance limits throughout the observation period.

A practical factor likely contributing to the observed stability is the aliquoting strategy used in this study. Repeated freeze–thaw cycles are known to affect analyte stability and may impair nucleic acid integrity, particularly in the case of RNA targets,^11^ while HIV RNA has also been shown to remain measurable under appropriate specimen handling conditions.^10^ Here, the controls were prepared as single‐use aliquots before storage, thereby minimizing repeated thawing and refreezing. This likely contributed substantially to the robust long‐term performance observed across all analytes. The findings therefore emphasize that appropriate pre‐analytical handling may be at least as important as storage duration itself within the time frame examined.

From a transfusion medicine perspective, these findings are relevant because NAT has become a central component of blood safety strategies and of the investigation of suspected transfusion‐transmitted infections.^4^ Although confirmed transmissions of HBV, HCV, and HIV are now rare in settings with established donor screening, retrospective molecular testing of stored specimens remains important for clarification of suspected cases and for quality assurance within hemovigilance systems. The present findings support confidence that archived frozen samples can retain adequate analytical performance for such purposes, provided that storage and handling conditions are appropriate.

The study adds to the limited available literature by providing long‐term longitudinal data from routine diagnostic practice on the quantitative performance of frozen virus‐positive plasma control material. In contrast to short‐term experimental storage studies, the present analysis reflects repeated measurement of the same control materials over extended periods under real‐world laboratory conditions, thereby offering a practice‐oriented perspective on long‐term analytical stability.

The study also highlights the value of patient‐derived diluted control materials for long‐term internal quality assurance in quantitative viral PCR. The very high proportion of results within mean1 ± 0.5 log10 indicates robust performance in routine use. More generally, the findings underscore the importance of continuous longitudinal review of quality control data in addition to reliance on an initial target value alone.^15^, ^16^

In addition, the use of patient‐derived internal run controls under routine diagnostic conditions represents a practical strength of the study, as it provides real‐world evidence on long‐term sample performance in a routine laboratory setting. This is particularly relevant because routine molecular diagnostics are subject to normal analytical variation over time, including potential assay‐related influences such as reagent lot changes, even if these factors were not systematically captured in the present retrospective dataset. Nevertheless, long‐term stability remained robust despite this real‐world scatter. Another strength is that the controls were prepared in the lower quantitative range of the respective assays, while remaining clearly above the manufacturer‐reported LOD/LLOQ. This makes the analysis particularly relevant for identifying potential loss of signal over time, as storage‐related effects would be expected to become apparent most readily in a range where quantitative fluctuations are generally more likely to occur. Several limitations should nevertheless be considered when interpreting these findings. First, this was a retrospective single‐center analysis based on routine quality control data generated under one defined laboratory setup. The study was designed to assess the long‐term stability of viral nucleic acids under routine analytical conditions and, at the same time, to document the robustness of the applied quantitative PCR platform during prolonged use of frozen positive control material. In addition, the IRCs analyzed in this study were generated by dilution of highly positive source material in negative plasma. Therefore, matrix‐ or dilution‐related effects cannot be completely excluded, and the findings should be extrapolated to original retained donor specimens with appropriate caution. In addition, this study did not evaluate original archived donor specimens with their full range of pre‐analytical variability, and this should be taken into account when interpreting the transferability of the findings to repository samples used in look‐back investigations. Factors such as sample collection setting, transport conditions, the interval between collection and freezing, and other donor‐related pre‐analytical variables may be relevant for repository donor samples but were outside the scope of the present study. However, because potentially relevant sources of routine analytical variation, such as reagent lot changes, were not systematically captured as separate variables, the findings should not be interpreted as a formal analysis of individual variance components and may not be directly transferable to other assay systems or laboratory workflows. Because acceptance criteria were defined on a log10 scale whereas temporal trend analyses were performed on raw quantitative values, minor differences in statistical significance may depend on the analytical scale chosen. However, the primary interpretation of long‐term stability was based on the predefined Rili‐BAEK acceptance criteria. Second, the storage conditions examined here corresponded to routinely monitored frozen storage in our laboratory (≤−18°C) and proved sufficient to preserve stable quantitative PCR performance over the periods studied.

In transfusion medicine, retained samples are stored at ≤−30°C; if storage temperature has any relevant influence on nucleic acid preservation, these lower temperatures would be expected to support at least comparable, if not more favorable, stability. Nevertheless, because storage specifications may vary between laboratories and regulatory frameworks, the present findings should be interpreted as evidence for stability under the specific conditions applied here rather than as a universal stability threshold. Third, no dedicated experimental assessment of repeated freeze–thaw stress, inter‐assay comparability, or viral subtype‐related effects was performed. Even so, the study provides practical evidence that long‐term frozen storage of HBV‐, HCV‐, and HIV‐positive plasma material can be compatible with stable quantitative PCR performance in routine diagnostics. This is relevant both for internal quality assurance and for retrospective molecular investigations when stored specimens need to be reanalyzed.

In conclusion, frozen HBV‐, HCV‐, and HIV‐positive samples demonstrated robust long‐term stability and reproducibility in routine quantitative PCR assays over periods of up to 4 years (49 months). Minor fluctuations and isolated outliers occurred, as expected in routine molecular diagnostics, but no statistically significant temporal decline was observed. These findings support the suitability of long‐term stored specimens for retrospective NAT and underline the importance of standardized aliquoting, frozen storage, and continuous quality monitoring.

## AUTHOR CONTRIBUTIONS

Conceptualization: Holger F. Rabenau. Methodology: Benjamin Marx, Annemarie Berger, Niko Kohmer, Marhild Kortenbusch, Sebastian Hoehl, Sandra Ciesek, and Holger F. Rabenau. Investigation: Benjamin Marx, Sandra Ciesek, and Holger F. Rabenau. Data curation: Benjamin Marx, Annemarie Berger, Niko Kohmer, Marhild Kortenbusch, and Sebastian Hoehl. Formal analysis: Benjamin Marx, Annemarie Berger, Niko Kohmer, Marhild Kortenbusch, Sebastian Hoehl, Sandra Ciesek, and Holger F. Rabenau. Visualization: Benjamin Marx, Annemarie Berger, and Holger F. Rabenau. Writing—original draft: Benjamin Marx, Annemarie Berger, Niko Kohmer, Marhild Kortenbusch, Sebastian Hoehl, Sandra Ciesek, and Holger F. Rabenau. Writing—review and editing: Benjamin Marx, Annemarie Berger, Niko Kohmer, Marhild Kortenbusch, Sebastian Hoehl, Sandra Ciesek, and Holger F. Rabenau. Supervision: Sandra Ciesek and Holger F. Rabenau. All authors reviewed the results and approved the final version of the article.

## FUNDING INFORMATION

No external funding was received for this study. The work was based on data generated during routine clinical and diagnostic practice.

## CONFLICT OF INTEREST STATEMENT

During Submission we declare that Annemarie Berger received Honorar and travel costs from Roche. All other Authors have nothing to declare.

## Data Availability

The data that support the findings of this study are available from the corresponding author upon reasonable request.

## References

[1] World Health Organization . WHO action framework to advance universal access to safe, effective and quality‐assured blood products 2020–2023. 2021. Available from: https://www.who.int/publications/i/item/9789240035744

[2] Alter HJ , Klein HG . The hazards of blood transfusion in historical perspective. Blood. 2008;112(7):2617–2626. 10.1182/blood-2008-07-077370 PMC2962447 18809775

[3] Busch MP , Kleinman SH , Nemo GJ . Current and emerging infectious risks of blood transfusions. JAMA. 2003;289(8):959–962. 10.1001/jama.289.8.959 12597733

[4] Perkins HA , Busch MP . Transfusion‐associated infections: 50 years of relentless challenges and remarkable progress. Transfusion. 2010;50(10):2080–2099. 10.1111/j.1537-2995.2010.02851.x 20738828

[5] Schreiber GB , Busch MP , Kleinman SH , Korelitz JJ . The risk of transfusion‐transmitted viral infections. The retrovirus epidemiology donor study. N Engl J Med. 1996;334(26):1685–1690. 10.1056/nejm199606273342601 8637512

[6] Stramer SL , Glynn SA , Kleinman SH , Strong DM , Caglioti S , Wright DJ , et al. Detection of HIV‐1 and HCV infections among antibody‐negative blood donors by nucleic acid‐amplification testing. N Engl J Med. 2004;351(8):760–768. 10.1056/NEJMoa040085 15317889

[7] Funk MB , Meyer B , Müller S . Hämovigilanz‐Bericht des Paul‐Ehrlich‐Instituts 2022: Auswertung der Meldungen von schwerwiegenden Reaktionen und Zwischenfällen nach § 63i AMG. 2025. Available from: https://pei.de

[8] Arbeitskreis Blut . Mitteilungen des Arbeitskreises Blut des Bundesministeriums für Gesundheit: Verfahren zur Rückverfolgung (Look Back) gemäß § 19 Transfusionsgesetz (Votum 48). Bundesgesundheitsblatt Gesundheitsforschung Gesundheitsschutz. 2021;64:108–125. 10.1007/s00103-020-03258-w

[9] Halfon P , Khiri H , Gerolami V , Bourliere M , Feryn JM , Reynier P , et al. Impact of various handling and storage conditions on quantitative detection of hepatitis C virus RNA. J Hepatol. 1996;25(3):307–311. 10.1016/s0168-8278(96)80116-4 8895009

[10] Sebire K , McGavin K , Land S , Middleton T , Birch C . Stability of human immunodeficiency virus RNA in blood specimens as measured by a commercial PCR‐based assay. J Clin Microbiol. 1998;36(2):493–498. 10.1128/jcm.36.2.493-498.1998 PMC104566 9466765

[11] Cuhadar S , Koseoglu M , Atay A , Dirican A . The effect of storage time and freeze‐thaw cycles on the stability of serum samples. Biochem Med (Zagreb). 2013;23(1):70–77. 10.11613/bm.2013.009 PMC3900085 23457767

[12] Ahmad‐Nejad P , Bauersfeld W , Baum H , Behre HM , Burkhardt R , Cassens U , et al. Revision of the “guideline of the German Medical Association on quality assurance in medical laboratory examinations—Rili‐BAEK”. J Lab Med. 2024;48(6):263–306. 10.1515/labmed-2024-0131

[13] Rabenau HF , Hofmann J , Schubert A , Hunfeld KP , Reischl U , Spitzenberger F . Die neue in‐vitro‐Diagnostika‐Verordnung (IVDR): Hilfestellung bei der Validierung/Verifizierung von im diagnostischen Laboratorium eingesetzten bzw. entwickelten und angewendeten Methoden zum Nachweis von Infektionserregern. Ger Med Sci. 2022;13: ISSN 1869‐4241. 10.3205/lab000044

[14] Rabenau HF , Kessler HH , Kortenbusch M , Steinhorst A , Raggam RB , Berger A . Verification and validation of diagnostic laboratory tests in clinical virology. J Clin Virol. 2007;40(2):93–98. 10.1016/j.jcv.2007.07.009 17766174

[15] Clinical and Laboratory Standards Institute . Statistical quality control for quantitative measurement procedures: principles and definitions. In: CLSI guideline. Wayne, PA: Clinical and Laboratory Standards Institute; 2006. p. C24.

[16] Westgard JO , Barry PL , Hunt MR , Groth T . A multi‐rule Shewhart chart for quality control in clinical chemistry. Clin Chem. 1981;27(3):493–501. 10.1093/clinchem/27.3.493 7471403

